# In Vitro Effects of Particulate Matter Associated with a Wildland Fire in the North-West of Italy

**DOI:** 10.3390/ijerph182010812

**Published:** 2021-10-14

**Authors:** Marta Gea, Sara Bonetta, Daniele Marangon, Francesco Antonio Pitasi, Caterina Armato, Giorgio Gilli, Fabrizio Bert, Marco Fontana, Tiziana Schilirò

**Affiliations:** 1 Department of Public Health and Pediatrics, University of Torino, 10126 Torino, Italy; marta.gea@unito.it (M.G.); cati.armato@gmail.com (C.A.); giorgio.gilli@unito.it (G.G.); fabrizio.bert@unito.it (F.B.); tiziana.schiliro@unito.it (T.S.); 2 Regional Agency for Environmental Protection of Piedmont (ARPA Piemonte), 10095 Grugliasco, Italy; daniele.marangon@arpa.piemonte.it (D.M.); francesco.pitasi@arpa.piemonte.it (F.A.P.); marco.fontana@arpa.piemonte.it (M.F.)

**Keywords:** forest fire, genotoxicity, mutagenicity, endocrine disruptors, particulate matter, air pollution, cytotoxicity, biological assays, BEAS-2B cells, MELN cells

## Abstract

Wildland fires, increasing in recent decades in the Mediterranean region due to climate change, can contribute to PM levels and composition. This study aimed to investigate biological effects of PM_2.5_ (Ø < 2.5 µm) and PM_10_ (Ø < 10 µm) collected near a fire occurred in the North-West of Italy in 2017 and in three other areas (urban and rural areas). Organic extracts were assessed for mutagenicity using Ames test (TA98 and TA100 strains), cell viability (WST-1 and LDH assays) and genotoxicity (Comet assay) with human bronchial cells (BEAS-2B) and estrogenic activity using a gene reporter assay (MELN cells). In all sites, high levels of PM_10_ and PM_2.5_ were measured during the fire suggesting that near and distant sites were influenced by fire pollutants. The PM_10_ and PM_2.5_ extracts induced a significant mutagenicity in all sites and the mutagenic effect was increased with respect to historical data. All extracts induced a slight increase of the estrogenic activity but a possible antagonistic activity of PM samples collected near fire was observed. No cytotoxicity or DNA damage was detected. Results confirm that fires could be relevant for human health, since they can worsen the air quality increasing PM concentrations, mutagenic and estrogenic effects.

## 1. Introduction

Wildland fires are complex phenomena influenced by numerous factors such as land management, human activities, and weather conditions [1].

All over the world climate change has been causing an increase in the frequency and intensity of extreme weather events [2] affecting also wildland fires. Indeed, the increase of occurrence of heatwaves and droughts, induced by climate change, has been promoting ignition and propagation of wildland fires and has also been causing effects on vegetation and forest fuels resulting in high risk of wildland fires [1]. The Mediterranean region is considered a high responsive area to global warming [3,4] and the combination of warmer and drier conditions will rise the risk of large fires characterized by an extensive burned area in the next decades [5,6].

Wildland fires induce both environmental and human health effects on firefighters as well as on the general population [7]. It was estimated that the exposure to wildland fire smoke causes each year a mortality equal to 339,000 deaths in the world [8,9]. Wildland fires can be associated with effects on the respiratory system (e.g. asthma and chronic obstructive pulmonary disease exacerbations, respiratory symptoms, decrease in lung function) [5,8]. However, exposure to wildland fires has been associated also with acute cardiovascular effects and birth outcomes [7,8]. 

Wildland fires can produce PM, aromatic hydrocarbons, aldehydes, metals, dioxins, furans, benzene, formaldehyde and other airborne pollutants [7,10,11]. However, the composition of produced smoke changes temporally and spatially according to the type of combustion and type of fuel [7,8,12,13]. 

The estimated emission of PM_2.5_ (Ø < 2.5 µm) from wildland fires is 0.9–16 g for each kg of burned dry biomass [10]. In South Europe as well as in South America, it was demonstrated that the release of PM by wildland fires, can significantly contribute to the airborne PM levels causing the exceeding of air quality standards [5,14,15,16]. These standards are set for PM regardless of its origin; however, the composition of wildland fire PM may be different from urban PM. Indeed, it has been demonstrated that particles from wood combustion have a higher content of total polycyclic aromatic hydrocarbons with respect to vehicle exhaust particles [17]. Moreover, different studies have shown that particles from biomass burning are predominantly composed of organic compounds and have lower metal concentrations than particles not deriving from biomass combustion [11,13]. These differences could lead to different biological effects. 

In order to evaluate the biological effects of PM, useful tools are represented by in vitro assays. They can inexpensively and quickly assess the total effect induced by a complex matrix, providing also information on the mechanism of action. In recent years, a growing concern has been focused on effects induced against the endocrine system and these effects have been studied using different in vitro assays such as estrogenic activity assays [18]. However, while numerous studies have applied these assays on waters and sediments [19], little is known about the estrogenic activity of airborne PM [20]. In contrast to the large amount of information on cytotoxicity, genotoxicity and mutagenicity induced by urban emissions [21,22], only few studies have evaluated biological effects induced by biomass combustion emissions during real wildland fires [23]; moreover, to our knowledge, the estrogenic activity of PM released from wildland fires has not yet been assessed. 

The aim of this study was to investigate different in vitro effects of PM collected during the wildland fire that occurred in October 2017 in Torino province (Piedmont region, North-West of Italy), an area characterized by critical air pollution levels due to geographical and meteorological conditions which promote the accumulation of air pollutants. PM_2.5_ (Ø < 2.5 µm) and PM_10_ (Ø < 10 µm) were collected near the wildland fire and in three other areas of Piedmont (two urban and one rural). Mutagenicity of organic extracts was assessed on different strains of *Salmonella typhimurium* (TA98, TA100) using Ames assay, while cell viability (WST-1 and LDH assays) and genotoxicity (Comet assay) were evaluated on human bronchial epithelial cells (BEAS-2B). Finally, in order to analyze the endocrine disrupting effects of PM, the estrogenic activity of organic extracts was estimated through a gene reporter assay on MELN cells.

## 2. Materials and Methods

### 2.1. Sampling and Organic Extraction of PM

In 2017 in Europe, wildland fires burnt over 1.2 million ha of natural lands; in particular, in Italy, there were 7855 fires, which burned 161,987 ha, the highest annual total burned area since 2007. The largest Italian wildland fire of the year occurred in Torino province (North-West of Italy) in autumn and covered 3533 ha [1].

**Figure 1:**
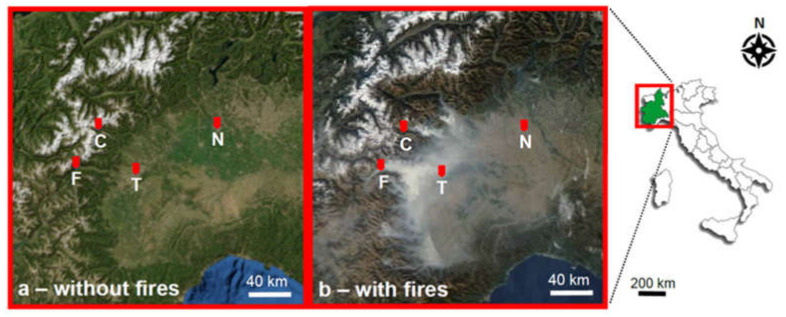
Geographical location of the monitoring stations. (**a**) Orthophotos, (**b**) Satellite image during wildland fires (25 October 2017) [24]. F = Chiomonte (wildland fire); T = Torino (high traffic); C = Ceresole Reale (rural); N = Novara (moderate traffic).

During October 2017, the Piedmont region was affected by numerous fires located in many valleys; among them, the widest wildland fire occurred in Chiomonte (Susa Valley, one of the sites of the XX Winter Olympic Games—Turin 2006). In this period, PM sampling was performed in the North-West of Italy through a mobile monitoring station and three monitoring stations of the Regional Agency for Environmental Protection of Piedmont (ARPA Piemonte) (Figure 1). The mobile station was located near the wide wildland fire of Chiomonte (rural village—site F). The other stations are permanently located: in a urban site characterized by high traffic level (city of Torino in the Padana Plain—site T), in a urban background site characterized by moderate traffic level (city of Novara in the Padana Plain—site N) and in a rural site (rural village of Ceresole Reale near the Gran Paradiso National Park—site C).

During the fire period, the Piedmont region was affected by a high number of days characterized by foehn wind, which contributed to the spread of fire [24]. Wind was mainly blowing from West–Southwest in site F (mean wind speed = 2.3 m/s, max wind speed = 17.9 m/s), causing the propagation of fire smoke from the fire site (F) to the Padana Plain (where are located site T and N) [24].

The four sites are characterized by different air pollution levels [25,26]. In the urban sites (T and N) the annual mean PM_10_ concentrations are higher than in the rural sites (F and C). Moreover, in urban sites a seasonal trend of air pollution is particularly evident: PM_10_ is higher in cold months and lower in warm months. This trend is characteristic of the study area, which is located in the Padana Plain. Here the geographical conformation together with the meteorological conditions promotes the accumulation of airborne pollutants, causing the exceeding of the WHO limits in particular during autumn-winter months [27,28,29,30,31]. 

PM_10_ (in the sites F, T, C) and PM_2.5_ (in the sites F, N) were sampled on quartz-fiber filters (Ø = 47 mm) with low volume sampler (flow 2.3 m^3^/h). For the mobile station (site F) PM filters were collected daily during the days characterized by the forest fire (from 17 October 2017 to 31 October 2017), while for the other stations (sites T, N, C) PM filters were collected daily during the whole October month (from 1 October 2017 to 31 October 2017). PM mass was estimated through a gravimetric method in compliance with the EN 12341 norm [32]. 

The daily filters were pooled to obtain one sample for each site (15 half filters for site F, 31 half filters for T, C, N sites) and each pool was extracted with Soxhlet (80 cycles) using acetone/hexane (1:1) in order to collect organic-extractable compounds. Extracts, evaporated with a rotary evaporator and re-suspended in dimethyl sulfoxide (DMSO), were stored at −20 °C until analysis.

### 2.2. Air Pollution Data

Air pollution data provided by the ARPA Piemonte were analysed in order to establish the effect of wildland fire on air quality in the different sites. In particular, the monthly mean of PM_10_ in the different sites was analysed from January 2016 to December 2018. Moreover, the concentration of specific tracers in PM for biomass burning emissions (levoglucosan, mannosan and galactosan) [33] during the wildland fire days was also considered. Data were only available for site F and site T. 

### 2.3. Cell Cultures 

BEAS-2B, human bronchial epithelial cells, were obtained from the American Type Culture Collection. BEAS-2B were grown and maintained in RPMI 1640 medium (supplemented with phenol red, fetal bovine serum (FBS) (10% *v*/*v*), L-glutamine (4 mM), penicillin-streptomycin (100 U/mL–100 µg/mL)), at 37 °C and 5% CO_2_. BEAS-2B were used to perform cytotoxicity and genotoxicity assays. Among the cell lines derived from the respiratory system, BEAS-2B were selected since they are non-tumoral derived cells characterized by normal growth and differentiation; moreover, they have been demonstrated to be a useful model for cell death and carcinogenesis studies [22]. 

MELN cells, provided by Dr. P. Balaguer (INSERM, Montpellier, France), are MCF-7 cells stably transfected with an estrogen-responsive gene (ERE-bGlob-Luc-SVNeo) carried by integrated plasmids. These plasmids contain both an antibiotic resistance selection gene (SVNeo) and the estrogen-responsive elements to which the estrogen receptor-ligand complex can bind, thereby inducing the transcription of the luciferase reporter gene. Therefore, the luciferase activity measured is proportional to the concentration of estrogenic compounds. MELN were grown and maintained in complete Dulbecco’s Modified Eagle’s Medium Nutrient Mixture F12-Ham (supplemented with phenol red FBS (5% *v*/*v*), L-glutamine (4 mM), penicillin-streptomycin (100 U/mL–100 µg/mL), G418 (1 mg/mL)), at 37 °C and 5% CO_2_. MELN were used to perform the luciferase gene reporter assay.

### 2.4. Cell Viability

Cell viability was assessed with WST-1 assay (Cell Proliferation Reagent WST-1, Roche) and lactate dehydrogenase release assay (LDH assay) (Cytotoxicity Detection Kit PLUS, Roche). The assays were performed as previously described by Gea et al. [34]. Briefly, BEAS-2B (70% confluent) were seeded in 24-well plates (4 × 10^4^ cells/well) and cultured overnight. Then, culture medium was replaced with organic extracts (equivalent to 0.5, 1, 2 m^3^/mL, see Table S1 in Supplementary Materials for the concentrations expressed as µg/mL) in RPMI medium without phenol red and cells were incubated for 24 h, 48 h or 72 h. The tested doses were selected in order to be representative of the real-life exposure.

For the WST-1 assay, after exposure, dye solution containing WST-1 was added (50 µL/well) and the cells were incubated for 2 h (37 °C, 5% CO_2_). Finally, the absorbance of each well was measured at 440 nm (Infinite Reader M200 Pro, Tecan). Cells exposed to the same amount of DMSO of the organic extract dilutions were used as negative control. The maximum percentage of DMSO tested was equal to 1%. Blank filter extract was also tested to verify that it did not induce any effect on cell viability.

For the LDH assay, after exposure the supernatant of each well (100 μL) was transferred into a clean well of a 96-well plate, mixed with Reaction Mixture (100 µL/well) and incubated for 30 min (room temperature). The reaction was interrupted with Stop Solution (50 µL/well) and the absorbance was measured at 490 nm (Infinite Reader M200 Pro, Tecan). Cells exposed to DMSO were used as negative control, while cells exposed to DMSO and lysed with Lysis Solution were used as positive control. 

In both assays, all experiments were performed in triplicate (three wells for each experimental condition) and data were expressed as a percentage of viability with respect to negative control (100%).

### 2.5. Genotoxicity 

The Comet assay was performed according to Tice et al. [35] with slight modifications [36]. BEAS-2B (70 % confluent) were cultured overnight in 6-well plates (3 × 10^5^ cells/well). Then, culture medium was replaced with organic extracts (equivalent to 0.5, 1, 2.5 m^3^/mL, see Table S1 in Supplementary Materials for the concentrations expressed as µg/mL) in base RPMI medium (without phenol red and without FBS) and cells were incubated for 24 h. The tested doses were selected in order to be representative of real-life exposure. After exposure, cell viability was determined (trypan blue staining) and cells were placed in low melting point agarose (0.7%) on slides. Slides were placed overnight in lysis solution (4 °C), were immersed in an alkaline electrophoresis buffer (20 min) and subjected to electrophoresis (20 min, 1 V/cm and 300 mA). Then, slides were neutralized, fixed and dried. For the analysis of DNA damage, cells in slides were stained with ethidium bromide (20 µg/mL) and the percentage of tail intensity was estimate using a fluorescence microscope (Axioskop HBO 50, Zeiss) equipped with the Comet Assay IV analysis system (Perceptive Instruments, Instem). Cells exposed to DMSO were used as negative control (maximum percentage of DMSO tested = 1%), while 4-nitroquinoline N-oxide (1, 1.5, 2 mg/L) was used as positive control. Blank filter extract was also tested to verify that it did not induce any DNA damage. All experiments were performed in duplicate (two gels for each experimental condition) and in each gel the % of tail intensity was quantified considering 50 cells. 

### 2.6. Ames Test 

In order to assess the mutagenicity, the Ames test was performed on each organic extract according to Maron and Ames [37]. Extracts were tested at different concentrations (2.5, 5, 10 and 20 m^3^/plate) in duplicate using two *Salmonella typhimurium* strains (frameshift strain-TA98 and base-substitution strain-TA100) both with and without Aroclor-induced rat-liver homogenate activation (±S9). 4-nitroquinoline 1-oxide (0.5 µg/plate), methyl methane-sulfonate (0.25 µg/plate) and 2-aminoanthracene (2 µg/plate) were used as positive controls for TA98, TA100 and TA98 + S9/TA100 + S9, respectively. DMSO was added to plates as negative control (100 µL/plate). Plates were incubated for 48 h and colonies were counted through an automatic colony counter (Synoptics Protos). 

The results were expressed as: (i) total revertants; (ii) mutagenicity ratio per 20 m^3^, MR [MR = (total revertants in plate exposed to 20 m^3^ − spontaneous revertants)/spontaneous revertants]; (iii) and total mutagenicity factor per 20 m^3^, TMF (TMF = MR TA98 + MR TA98 (+S9) + MR TA100 + MR TA100 (+S9)). For each strain, with or without S9, the mutagenic effect was considered significant when mean of total revertants were at least twice than mean of spontaneous revertants (MR ≥ 1), while an extract was considered mutagen when at least one strain, with or without S9, showed an MR ≥ 1. Blank filter extract was also tested to verify that it did not induce any mutagenic effect.

### 2.7. Estrogenic Activity

Estrogenic activity was assessed with the luciferase gene reporter assay using the One-Glo Luciferase Assay System (Promega). The assay was carried out as described by Balaguer et al. [38] with some modifications [39]. For three days cells were cultured in test medium (Dulbecco’s Modified Eagle’s Medium Nutrient Mixture F12-Ham supplemented with dextran-coated charcoal-treated FBS (5% *v*/*v*), L-glutamine (4 mM), penicillin-streptomycin (100 U/mL–100 µg/mL)). MELN (70% confluent) were seeded in 96-well plates with a flat clear bottom (4 × 10^4^ cells/well) and cultured overnight. The day after, test medium was replaced with organic extracts (equivalent to 0.5, 1, 2 m^3^/mL, see Table S1 in Supplementary Materials for the concentrations expressed as µg/mL) in test medium and cells were incubated for 20 h. At the end of the incubation, One-Glo Reagent (100 µL/well) was added and, after 5 minutes, the luminescence was measured by a luminometer (Infinite Reader M200 Pro, Tecan). Cells exposed to DMSO were used as negative control (maximum percentage of DMSO tested = 1%) and five concentrations of 17β-estradiol (range 10^−12^–10^−8^ M) were tested as a standard positive curve. The extracts (1 m^3^/mL) were also tested in combination with tamoxifen (10^−6^ M) in order to confirm whether the effects observed were due to activation of estrogen-receptors (ERs) and in combination with 17β-estradiol (10^−10^ M) to test the interaction between each extract and 17β-estradiol. Blank filter extract was also tested to verify that it did not induce any estrogenic activity.

The luciferase activity was expressed as fold induction with respect to negative control (fold induction = 1). All experiments were performed in quadruplicate (four wells for each experimental condition).

### 2.8. Statistical Analysis 

Statistical analysis was performed using SPSS 26.0 (IBM Statistics, Armonk, NY, USA). The *t*-test and the one-way ANOVA test followed by post-hoc Dunnett test were used to assess significant differences between effects induced by extracts and effects induced by negative controls. Moreover, the *t*-test was used to assess significant differences among the mutagenic effects induced by the extracts collected in different sites. The differences were considered significant for *p* < 0.05.

## 3. Results and Discussion

### 3.1. Air Pollution Data

In Table 1 are reported the mean PM concentrations in the four sites during the sampling period. All PM concentrations were above the daily guideline values set by the WHO [40], except for the site C. These high values may be partially due to the increase of PM levels due to wildland fire but also to other PM sources such as traffic, home heating, and industrial processes especially in urban sites (T and N).

In order to establish whether the high values of PM in the different sites were influenced by the wildland fire, for each site the monthly means of PM_10_ were analysed from January 2016 to December 2018. 

**Figure 2:**
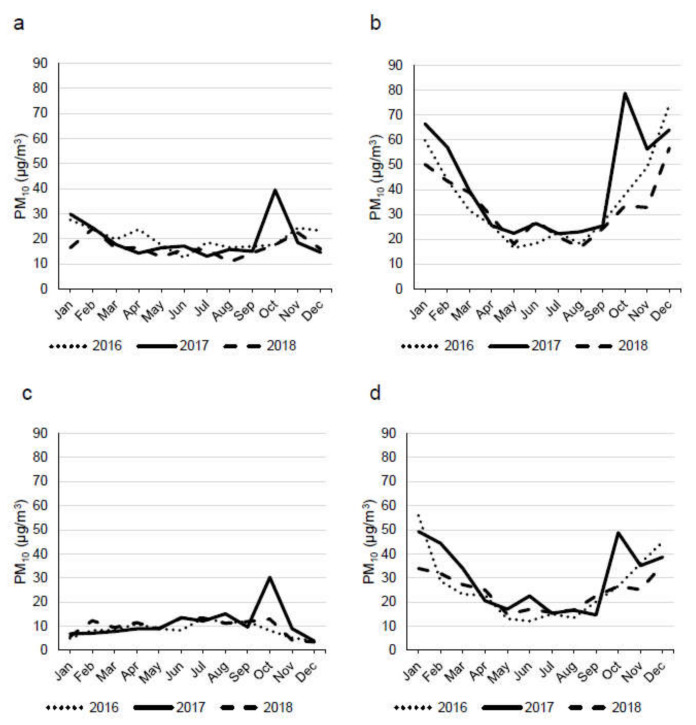
Monthly mean of PM_10_ concentrations in the four sampling sites: Chiomonte—site F (**a**), Torino—site T (**b**), Ceresole Reale—site C (**c**), Novara—site N (**d**). Wildland fire period = October 2017.

As can be seen in Figure 2, despite the different pollution levels of the four sampling sites, in all the sites a particular high level of PM_10_ was registered during the wildland fire month (October 2017). This evidence suggests that not only the sites near the wildland fire but also distant sites, such as N, were probably influenced by the air pollutants associated with the wildland fire.

The influence of the wildland fire on the air quality of rural and urban areas was further investigated considering the concentrations of specific biomass burning tracers in PM [33]. In particular, the concentrations of levoglucosan, mannosan and galactosan in the PM_10_ were selected as biomass burning tracers, because, during combustion, large quantities of these compounds are emitted in the smoke aerosol. The daily concentrations of these three tracers in the rural site F (near fire) and in the urban site T are reported in Supplementary Materials Figure S1. In the wildland fire site (F) as well as in the urban site (T) there is an increase of the concentrations starting from the 23 October for all the biomass burning tracers, corresponding with the days characterized by the most intense burning. It is interesting to highlight that, for all the tracers, the concentrations increase earlier in the site F (near the wildland fire) with respect to the urban site T suggesting the presence of a temporal shift of the air pollution levels. This shift could be due to the time needed for the air pollutants released by the fire to reach the urban site.

Taking together, the air pollution data confirm that wildland fires are associated with an increase of PM as reported by previous studies [41,42,43,44,45]. Moreover, these data suggest that the air pollutants released by wildland fires can influence the air quality in the nearby area but also in many surrounding areas. 

### 3.2. Cell Viability and Genotoxicity

Since the airborne pollution induced by wildland fires can mainly affect the respiratory system, the cell viability and the genotoxicity were investigated using human bronchial epithelial cells (BEAS-2B).

Despite the different doses and the different exposure times, the extracts did not induce any cytotoxic effect in the WST-1 assay (*t*-test vs. negative control *p* > 0.05, Supplementary Materials Figure S2). Using the LDH assay, no cytotoxicity was detected (*t*-test vs. negative control *p* > 0.05, Supplementary Materials Figure S3), confirming that all the extracts did not induce a decrease in cell viability with respect to negative control. These results are consistent with those of De Oliveira Galvao et al. [46], who demonstrated that PM_10_ organic extracts collected in the Brazilian Amazon during wildland fires did not induce any cytotoxic effect in human alveolar epithelial cells (A549). However, these results differ from other published studies [41,42,45,47] in which a significant decrease in cell viability was detected exposing cells (A549 or RAW 264.7) to PM produced by wildland fires.

Moreover, all the PM extracts did not induce any genotoxic effect measured as DNA damage (One-way ANOVA test followed by post-hoc Dunnett test vs. negative control *p* > 0.05, Supplementary Materials Figure S4), while in previous in vitro studies a significant genotoxic effect was induced by PM released from wildland fires [41,46,48].

The different results obtained in this study with respect to previous studies could be due to differences in PM composition which is influenced by the different characteristics of wildland fire (e.g. extension, type of combustion, type of vegetation burned) [7,8,12,13]. However, it is also important to highlight that in the present study low doses of PM, representative of the real exposure, were tested. 

### 3.3. Ames Test 

**Figure 3:**
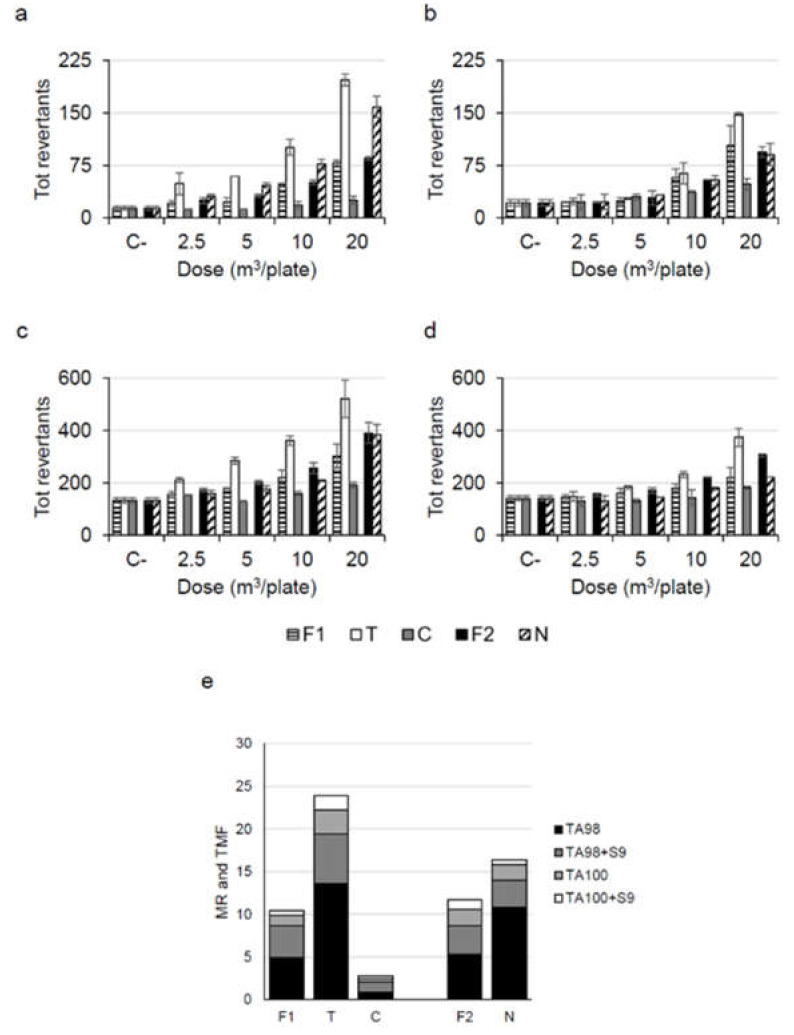
Mutagenicity detected in PM extracts using different *Salmonella typhimurium* strains with and without metabolic activation (± S9). Data are expressed as: total revertants and mutagenicity ratio in 20 m^3^ of air (MR). TMF = Total Mutagenicity Factor (∑MR); F1 = PM_10_ extract from Chiomonte—site F (rural site, fire site); T = PM_10_ extract from Torino—site T (urban site); C = PM_10_ extract from Ceresole Reale—site C (rural site); F2 = PM_2.5_ extract from Chiomonte—site F (rural site, fire site); N = PM_2.5_ extract from Novara—site N (urban site). (**a**): total revertants—strain TA98, (**b**): total revertants—strain TA98 with metabolic activation (+S9), (**c**): total revertants—strain TA100, (**d**): total revertants—strain TA100 with metabolic activation (+S9), (**e**): MR and TMF—all strains with and without metabolic activation (±S9).

The Ames test using different *Salmonella typhimurium* strains (TA98, TA100) with and without metabolic activation (±S9) was performed to assess the mutagenicity of the PM organic extracts. The results are presented in Figure 3.

For both the strains, the effect was higher without metabolic activation (−S9), suggesting that the mutagenicity was mainly due to the presence of direct mutagenic substances. Moreover, the extracts induced a higher mutagenicity on TA98 than on TA100 strain, hence the mutagenic effect was induced mainly by frame-shift mutations instead of replacement of nitrogenous bases. 

Considering the results obtained on TA98 strain, the samples collected in the two urban sites (T and N) induced a number of total revertants that was at least twice than mean spontaneous revertants for all tested doses (from 2.5 to 20 m^3^/plate, Figure 3a), therefore a significant mutagenic effect was detected for all tested doses in these urban sites. On the contrary, the rural sites (F and C) induced a significant effect starting from higher doses. At 20 m^3^/plate the urban extracts induced a significantly higher number of TA98 revertants than the rural extracts (number of TA98 revertants at 20 m^3^/plate *t*-test significant for: T vs. F1 *p* = 0.014, T vs. C *p* = 0.003, N vs. F2 *p* = 0.022). This result is in accordance with a recent study in which urban and rural samples (collected in the Piedmont region) were analyzed using the Ames test [49]. Indeed, Marangon et al. [49] also found a higher mutagenicity for the urban sites with respect to the rural site during autumn-winter months. The higher mutagenic effect of the urban sites than of the rural sites could be due to the higher content of mutagenic substances in urban areas and also to the higher PM concentration in these areas with respect to rural areas. Considering the results obtained on TA98 + S9, TA100 and TA100 + S9, a lower mutagenic effect was detected with respect to TA98. Moreover, in these experimental conditions, a higher effect was induced by the first urban extract (T site), while the mutagenic effect induced by the second urban extract (N site) was more similar to the effect induced by the rural extracts (F and C sites).

Overall, all the extracts were mutagenic; however, comparing the mutagenic effect of the different sites, the highest TMF was induced by the PM_10_ collected in the site T, an urban site characterized by high traffic levels (Figure 3e). The mutagenicity induced by this extract was particularly strong with respect to mutagenicity generally measured in this site in other years during October. Indeed, the TMFs of the PM_10_ organic extracts collected in the site T during October 2016 and October 2018 were less than a half of the TMF of the PM_10_ collected in the same site during October 2017 (site T TMF = 23.9—October 2017; TMF = 8.6—October 2016, TMF = 9.2—October 2018). This result confirmed the influence of the wildland fire on the air quality of this site.

Regarding the other PM_10_ extracts, the rural site located near the fire (F1) induced a less mutagenic effect than the organic extract collected in the site T but a more mutagenic effect than the extract collected in the other rural site (C) (number of TA98 revertants at 20 m^3^/plate *t*-test significant for: F1 vs. T *p* = 0.014, F1 vs. C *p* = 0.013). This result suggests that the rural site near the fire may be influenced by some mutagenic substances released by the fire itself that are responsible for the higher mutagenic effect with respect to the effect induced by the other rural site, located more distantly from fires. It is important to highlight that the organic extract collected near the fire (F1) induced a mutagenic effect which was similar to the effect measured in the site T in a period not characterized by fires (site F1 TMF = 10.4, site T TMF = 8.6, October 2016, site T TMF = 9.2, October 2018), suggesting that the fire was able to deteriorate the air quality of this rural site. Moreover, it is also important to notice that, although the mutagenicity measured in C was low, the presence of this effect is unexpected in this site which is located far from big cities/industrial facilities and near the Gran Paradiso National Park, suggesting an influence of the fires on this site also. 

As reported for PM_10_, the mutagenicity of PM_2.5_ organic extracts showed that the urban site N induced a higher effect than the site F (F2) (number of TA98 revertants at 20 m^3^/plate *t*-test significant for: N vs. F2 *p* = 0.022). However, the difference of the mutagenic effect between the urban sites and the fire site was higher for PM_10_ extracts than for PM_2.5_ extracts (T TMF—F1 TMF higher than N TMF—F2 TMF). This result could be explained considering that the two urban PM samples were collected in different cities, which may be characterized by different pollution levels [28]; however, this difference could be also due to the different contribution of the fire released pollutants in the different cities. Indeed, Torino (T) is located closer to the fires with respect to Novara (N). 

Overall, the results of the Ames test are consistent with the air pollution data and confirm that the wildland fires are able to influence the air quality of the sites near the wildland fire (site F) but also of distant sites (i.e. site C and site T). 

Comparing at equal doses the results of the Ames test with the results of the Comet assay, a significant mutagenic effect was detected in the Ames test (strain TA98, dose = 2.5 m^3^/plate, site T and site N), while no significant genotoxic effect, measured as DNA damage, was observed with the Comet assay (dose = 2.5 m^3^/mL) (One-way ANOVA test followed by post-hoc Dunnett test *p* > 0.05). These results are consistent with the study of De Oliveira Galvão et al. [46], in which, testing wildland fire PM extracts, a higher effect was measured using the Ames test with respect to the effect measured using a genotoxicity test on mammalian cells (the cytokinesis-block micronucleus test). Moreover, they are consistent also with other studies on PM_0.5_, PM_2.5_ and PM_10_ extracts which reported a higher effect measured using the Ames test with respect to the effect measured using the comet assay on mammalian cells [36,50,51]. The different biological effects that were found using the two assays may be related to the fact that the test methods address different genetic endpoints; moreover, bacteria and mammalian cells show differences in chemical accessibility, in metabolism and in toxicity (e.g. cell wall vs. plasma membrane, lower vs. higher antioxidant activity, stress induced response consisting of mutation vs. cell death, DNA damage repair aimed at survival/mutation vs. repair aimed at fidelity) [52].

### 3.4. Estrogenic Activity

To our knowledge, this is the first study in which the estrogenic activity of PM released from wildland fires was assessed. Indeed, there are still few studies on estrogenic activity of PM [20,53,54] and no one has investigated this specific effect during wildland fires. However, among the numerous pollutants generated by biomass combustion, some might alter the function of the endocrine system (e.g. polycyclic aromatic hydrocarbons, dioxins, dibenzofurans) and thus they can be considered as endocrine disrupting chemicals (EDCs) [55]. EDCs can affect different hormones and many of them can interfere with the endocrine system through the binding with estrogen receptors (ERs) causing an agonist activity (estrogenic pollutants) or an antagonist activity (anti-estrogenic pollutants) [18,56].

**Figure 4:**
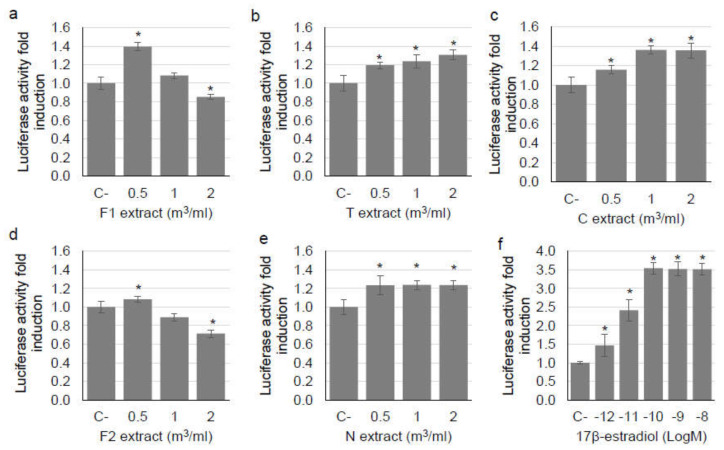
Estrogenic activity detected using luciferase gene reporter assay in PM_10_ extracts collected in (**a**) Chiomonte (wildland fire)—F1, (**b**) Torino—T, (**c**) Ceresole Reale—C and in PM_2.5_ extracts collected in (**d**) Chiomonte (wildland fire)—F2, (**e**) Novara—N. The results are expressed as luciferase activity fold induction (means and standard deviations) respect to negative control (C- = 1). (**f**) standard positive curve, obtained exposing cells to five concentrations of 17β-estradiol (10^−12^–10^−8^ M). * *p* < 0.05 vs. C- according to *t*-test.

The results of the luciferase gene reporter assay are presented in Figure 4.

The PM_10_ and PM_2.5_ extracts collected near the wildland fires (F1 and F2), showed a significant increase of luciferase activity with respect to negative control only at 0.5 m^3^/mL (*t*-test *p* < 0.0001, *p* = 0.011, respectively) while a significant decrease of luciferase activity was detected at 2 m^3^/mL (*t*-test *p* = 0.023, *p* = 0.001, respectively). On the contrary, the PM collected in the sites T, C and N induced an increase of luciferase activity which was significant for all the tested doses (*t*-test *p* < 0.01 for all sites and doses), suggesting a higher estrogenic activity with respect to the wildland fire extracts. 

Overall, the extracts showed low increases of luciferase activity with respect to negative control and thus a low estrogenic activity. Indeed, the maximum observed effect was induced by PM_10_ collected in F (wildland fire) and was equal to 1.39 luciferase activity fold induction (Figure 4a), while the standard positive curve showed a maximum effect equal to 3.54 (Figure 4f). The estrogenic activity of PM extracts, despite being low, is consistent with other studies in which an estrogenic activity was detected in PM extracts collected outdoor in urban and rural sites [20,53,57,58,59].

In the present study, it is interesting to notice that while the estrogenic activity of urban and rural sites increases or remains constant with the increase of the dose, the estrogenic activity of wildland fire (both PM_10_ and PM_2.5_) seems to show a non-monotonic dose response (i.e. an inverted-U shaped curve). This result might be related to a cytotoxic effect/cell proliferation inhibition induced by the extracts on MELN [60]; however, this hypothesis is not supported by the cell viability results since no cytotoxic effect was observed on BEAS-2B exposed to same doses also for longer exposure times (48 h, 72 h). Hence this result might be explained considering that many EDCs shows a non-monotonic dose response profile which can be clarified by other hypothesis such as the presence of different receptors with different affinities to EDCs and opposite effects (i.e. agonist or antagonist), negative feed-back phenomena, high-dose receptor desensitization [60]. In particular, this non-monotonic profile could be due to the presence of some pollutants which, at specific concentrations, can exert an estrogen antagonist activity. For example, dioxins and polycyclic aromatic hydrocarbons are released by wildland fires [55,61] and can exert estrogen antagonist activity [55]. Therefore, the results observed in the present study for F1 and F2 might be due to antagonist effects induced by dioxins and polycyclic aromatic hydrocarbons at the highest concentrations tested (1, 2 m^3^/mL). 

This hypothesis was further investigated testing the estrogenic activity of all the extracts (1 m^3^/mL) in combination with E2 (10^−10^ M). The PM collected in urban and rural sites tested with E2 (T + E2, C + E2, N + E2) showed a luciferase activity similar to the activity induced by the E2 alone (*t*-test *p* > 0.05), while the wildland fire PM tested with E2 (F1 + E2, F2 + E2) showed a significantly lower luciferase activity with respect to the E2 alone (*t*-test *p* < 0.0001 for both samples, Supplementary Materials Figure S5). This result suggests a possible antagonist effect of wildland fire PM when tested in combination with E2. An antagonistic effect of PM was previously reported also by other authors for PM samples [54,57,62].

Further studies are needed to assess the estrogenic activity of PM collected during wildland fires; indeed the data on estrogenic activity of PM is still limited and, in this area, no estrogenic activity data collected during periods without fires are available. Moreover, to our knowledge, no study has investigated the estrogenic activity of PM during wildland fires, therefore it is not possible to compare these results with others. Future studies should be performed assessing the estrogenic activity of PM in urban and rural areas of the North-West of Italy without wildland fires in order to obtain useful data for comparison. For the same reason, in future studies it may be useful to carry out a first sampling campaign in sites affected by wildland fires and also a second sampling campaign a year later in the same areas during the same period. Moreover, for future studies, areas located even more distant than the wildland fire site should be considered, in order to collect data in sites not influenced by wildland fires. Finally, in future studies, a higher amount of each sample should be collected in order to have enough extract for the assessment of the biological effects, also using higher concentrations than the concentrations tested in this study. Moreover, the sampling of many m^3^ of air in each site could also allow to perform a chemical characterization of the extracts, providing additional and complementary information. 

## 4. Conclusions

The occurrence of wildland fires is growing due to climate change and specific regions, such as the Mediterranean area, will probably be more affected than others. During wildland fires, numerous hazardous pollutants are released in the atmosphere, representing a possible threat for human health. Since the increase of knowledge on air pollution induced by wildland fires is needed, it is of fundamental importance to investigate the effects induced by PM collected during wildland fire. The results of this study suggest that wildland fires worsen the air quality, increasing the concentrations of air pollutants, such as PM, and the mutagenic effect of the PM. This effect seems to influence the sites near fires, but also distant sites. Therefore fires, even if they are sporadic and short-time episodes, should be considered important human health issues. Moreover, in this study, the preliminary results on the estrogenic activity induced by PM collected during wildland fires highlighted some research gaps that have to be further investigated.

## Figures and Tables

**Table 1:** Characteristics and PM concentrations of the sampling sites.

Site			PM		
**Location**	Acronym	Characteristics	Diameter (µm)	Concentration ± SD (µg/m^3^) ^#^	Daily Guideline Values (µg/m^3^) *
**Chiomonte**	F1	Rural (wildland fire)	10	51 ± 39	50
**Torino**	T	Urban high traffic		79 ± 46	
**Ceresole Reale**	C	Rural		30 ± 39	
**Chiomonte**	F2	Rural (wildland fire)	2.5	43 ± 34	25
**Novara**	N	Urban back-ground		36 ± 21	

^#^ monthly mean concentration (October 2017) for T, C, N; mean concentration from 17 October 2017 to 31 October 2017 for F. * according to WHO guideline values (2006).

## Data Availability

Data sharing not applicable.

## Supplementary Materials

The following are available online at https://www.mdpi.com/article/10.3390/ijerph182010812/s1, Table S1: Concentrations of PM tested in the biological assays expressed as m^3^/mL and µg/mL. F1—PM_10_ collected in Chiomonte (wildland fire); T—PM_10_ collected in Torino; C—PM_10_ collected in Ceresole Reale; F2—PM_2.5_ collected in Chiomonte (wildland fire); N—PM_2.5_ collected in Novara; Figure S1: Daily concentrations of biomass burning tracers in the rural site F (Chiomonte) and in urban site T (Torino): levoglucosan concentrations (a), mannosan concentrations (b), galactosan concentrations (c). Data are compared with the PM_10_ concentrations measured in the same sites (PM_10_ concentrations are not available for 17th and 21st October 2017 in site F); Figure S2: Cell viability detected using WST-1 assay in PM_10_ extracts collected in (a) Chiomonte (wildland fire), (b) Torino, (c) Ceresole Reale and in PM_2.5_ extracts collected in (d) Chiomonte (wildland fire), (e) Novara. Data expressed as % of cell viability (means and standard deviations) with respect to negative control (C- = 100%). T-test was not significant (data vs. C-, *p* > 0.05); Figure S3: Cell viability detected using LDH assay in PM_10_ extracts collected in (a) Chiomonte (wildland fire), (b) Torino, (c) Ceresole Reale and in PM_2.5_ extracts collected in (d) Chiomonte (wildland fire), (e) Novara. Data expressed as % of cell viability (means and standard deviations) with respect to negative control (C- = 100%). T-test was not significant (data vs. C-, *p* > 0.05); Figure S4: DNA damage detected using Comet assay in PM_10_ extracts collected in (**a**) Chiomonte (wildland fire)—F1; (**b**) Torino—T; (**c**) Ceresole Reale—C and in PM_2.5_ extracts collected in (**d**) Chiomonte (wildland fire)—F2 (**e**) Novara—N. Data expressed as % of tail intensity (means and standard deviations). One-way ANOVA test followed by post-hoc Dunnett test not significant (data vs. C-, *p* > 0.05); Figure S5: Estrogenic activity detected using luciferase gene reporter assay in the PM extracts (1 m^3^/mL) tested in combination with 17β-estradiol (10^−10^ M). The results are expressed as luciferase activity fold induction (means and standard deviations) respect to negative control (C- = 1). E2 = 17β-estradiol (10^−10^ M). * *p* < 0.05 vs. C- according to *t*-test. # *p* < 0.05 vs. E2 according to *t*-test.
