# Supplementary material for: In Vitro Effects of Particulate Matter Associated with a Wildland Fire in the North-West of Italy
Source: Int J Environ Res Public Health. 2021 Oct 14;18(20):10812. doi: 10.3390/ijerph182010812 (PMC8535364; doi:10.3390/ijerph182010812)
Supplement: Supplementary file 1 [file ijerph-18-10812-s001.zip › ijerph-1401780-SI.pdf]

International Journal of *Environmental Research and Public Health*

SUPPLEMENTARY MATERIALS

In vitro effects of particulate matter associated with a wildland fire in the North-West of Italy

Marta Gea[1], Sara Bonetta[1*], Daniele Marangon[2], Francesco Antonio Pitasi[2], Caterina Armato[1], Giorgio Gilli[1], Fabrizio Bert[1], Marco Fontana[2], Tiziana Schilirò[1]

[1] Department of Public Health and Pediatrics, University of Torino, Piazza Polonia 94, 10126 Torino, Italy.

[2] Regional Agency for Environmental Protection of Piedmont (ARPA Piemonte), 10135 Torino, Italy.

***Corresponding author**:

Corresponding author:

Sara Bonetta

sara.bonetta@unito.it

Department of Public Health and Pediatrics,

University of Torino,

Piazza Polonia 94, 10126 Torino, Italy.

Phone: +39 011 6708192

CONTENTS:

Table S1
Figure S1
Figure S2
Figure S3
Figure S4
Figure S5

Submitted to ***INTERNATIONAL JOURNAL OF ENVIRONMENTAL RESEARCH AND PUBLIC HEALTH***

SUPPLEMENTARY MATERIALS

Table S1. Concentrations of PM tested in the biological assays expressed as m^3/ml and µg/ml. F1 – PM_{10} collected in Chiomonte (wildland fire); T – PM_{10} collected in Torino; C – PM_{10} collected in Ceresole Reale; F2 – $PM_{2.5}$ collected in Chiomonte (wildland fire); N – $PM_{2.5}$ collected in Novara.

		**m³/ml**	**µg/ml**
**F1**	dose 1	0.5	25.5
	dose 2	1.0	51.0
	dose 3	2.0	102.0
	dose 4	2.5	127.5
**T**	dose 1	0.5	39.5
	dose 2	1.0	79.0
	dose 3	2.0	158.0
	dose 4	2.5	197.5
**C**	dose 1	0.5	15.0
	dose 2	1.0	30.0
	dose 3	2.0	60.0
	dose 4	2.5	75.0
**F2**	dose 1	0.5	21.5
	dose 2	1.0	43.0
	dose 3	2.0	86.0
	dose 4	2.5	107.5
**N**	dose 1	0.5	18.0
	dose 2	1.0	36.0
	dose 3	2.0	72.0
	dose 4	2.5	90.0

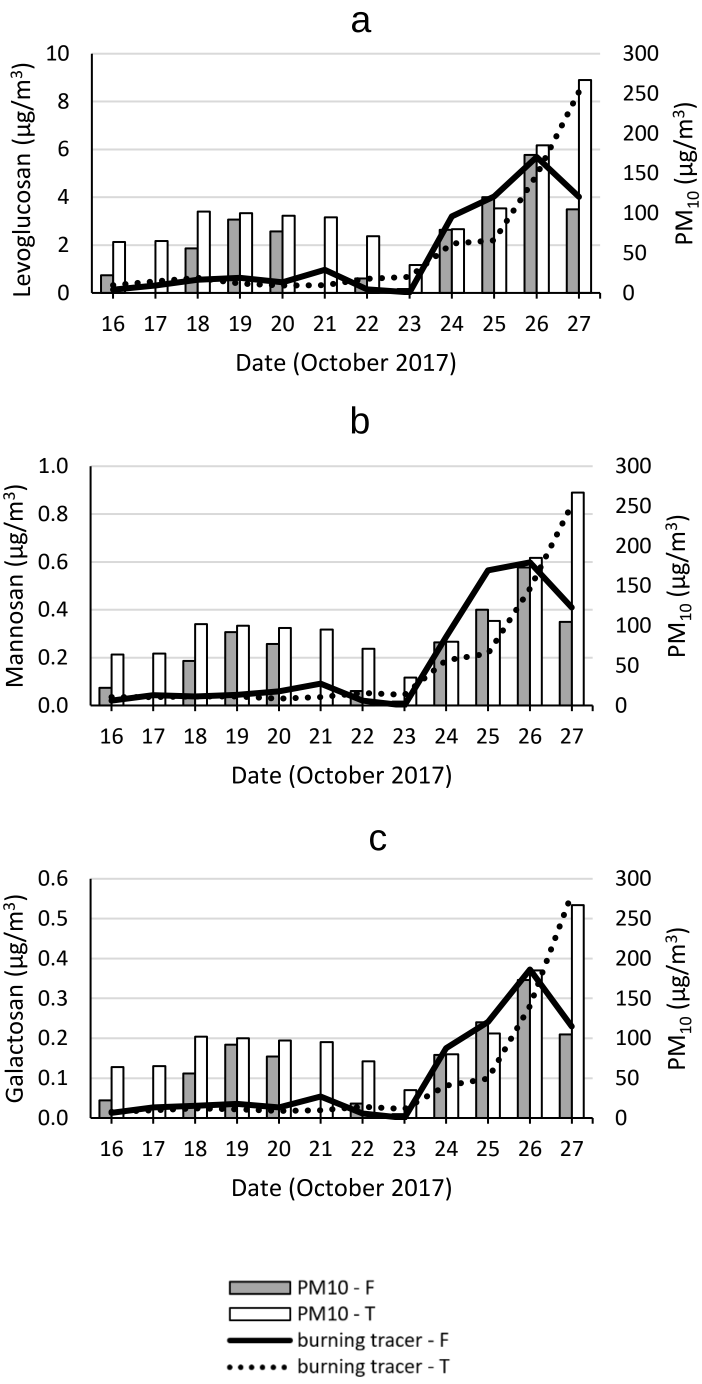

Figure S1. Daily concentrations of biomass burning tracers in the rural site F (Chiomonte) and in urban site T (Torino): levoglucosan concentrations (**a**), mannosan concentrations (**b**), galactosan concentrations (**c**). Data are compared with the PM_{10} concentrations measured in the same sites (PM_{10} concentrations are not available for 17th and 21st October 2017 in site F).

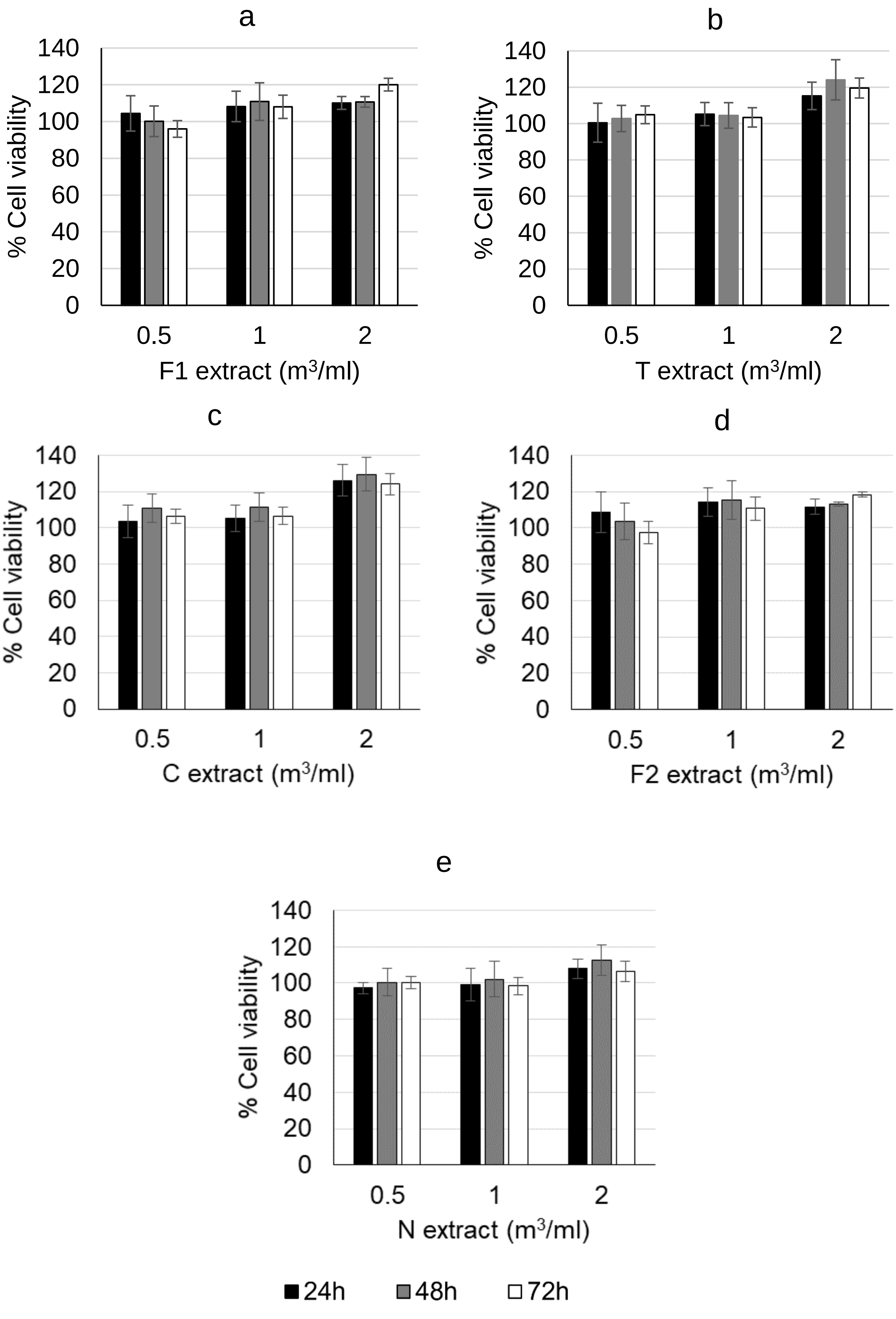

Figure S2. Cell viability detected using WST-1 assay in PM_{10} extracts collected in (**a**) Chiomonte (wildland fire), (**b**) Torino, (**c**) Ceresole Reale and in $PM_{2.5}$ extracts collected in (**d**) Chiomonte (wildland fire), (e) Novara. Data expressed as % of cell viability (means and standard deviations) with respect to negative control (C- = 100%). T-test was not significant (data *vs* C-, $p > 0.05$).

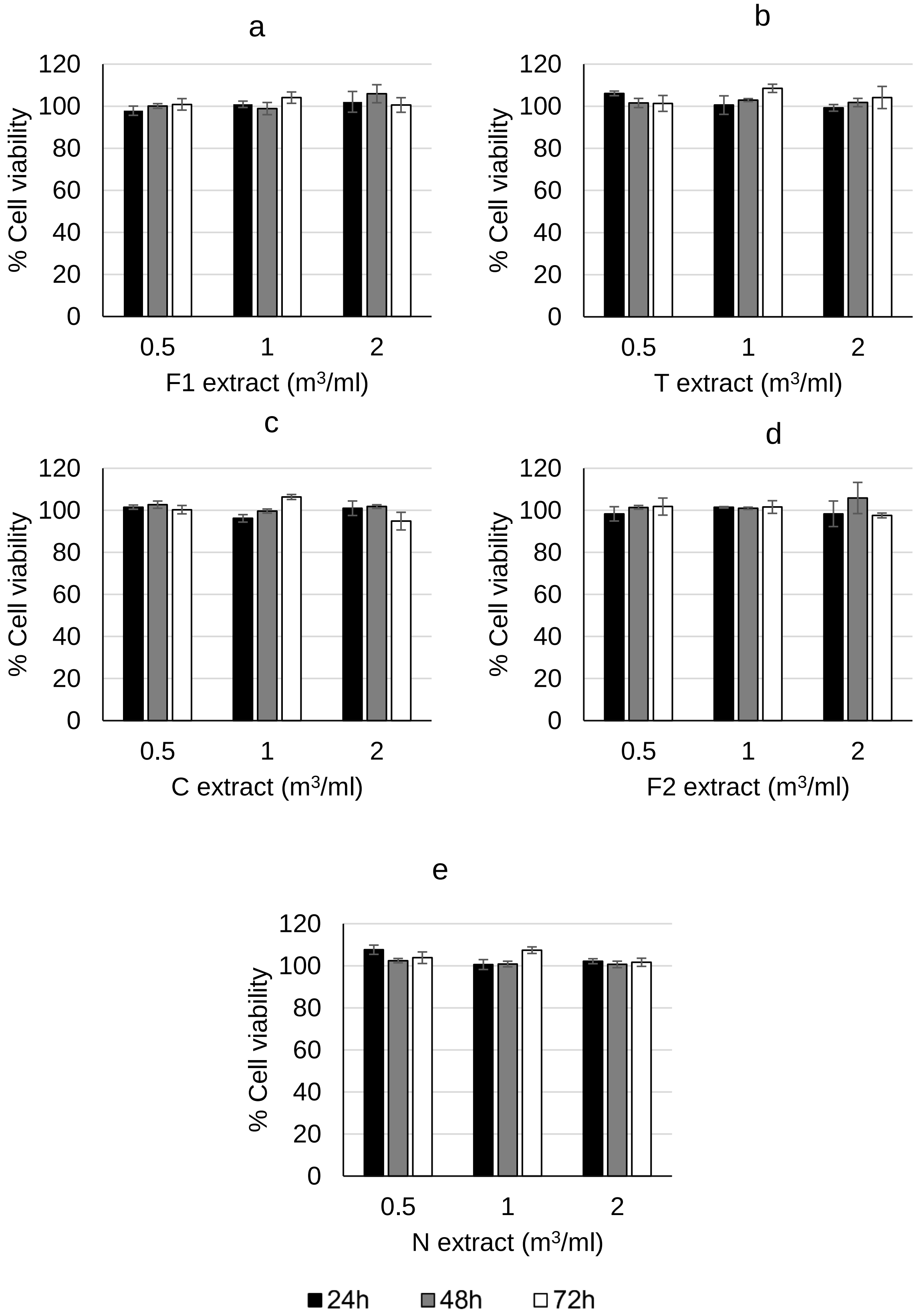

Figure S3. Cell viability detected using LDH assay in PM_{10} extracts collected in (**a**) Chiomonte (wildland fire), (**b**) Torino, (**c**) Ceresole Reale and in $PM_{2.5}$ extracts collected in (**d**) Chiomonte (wildland fire), (e) Novara. Data expressed as % of cell viability (means and standard deviations) with respect to negative control (C- = 100%). T-test was not significant (data *vs* C-, $p > 0.05$). .

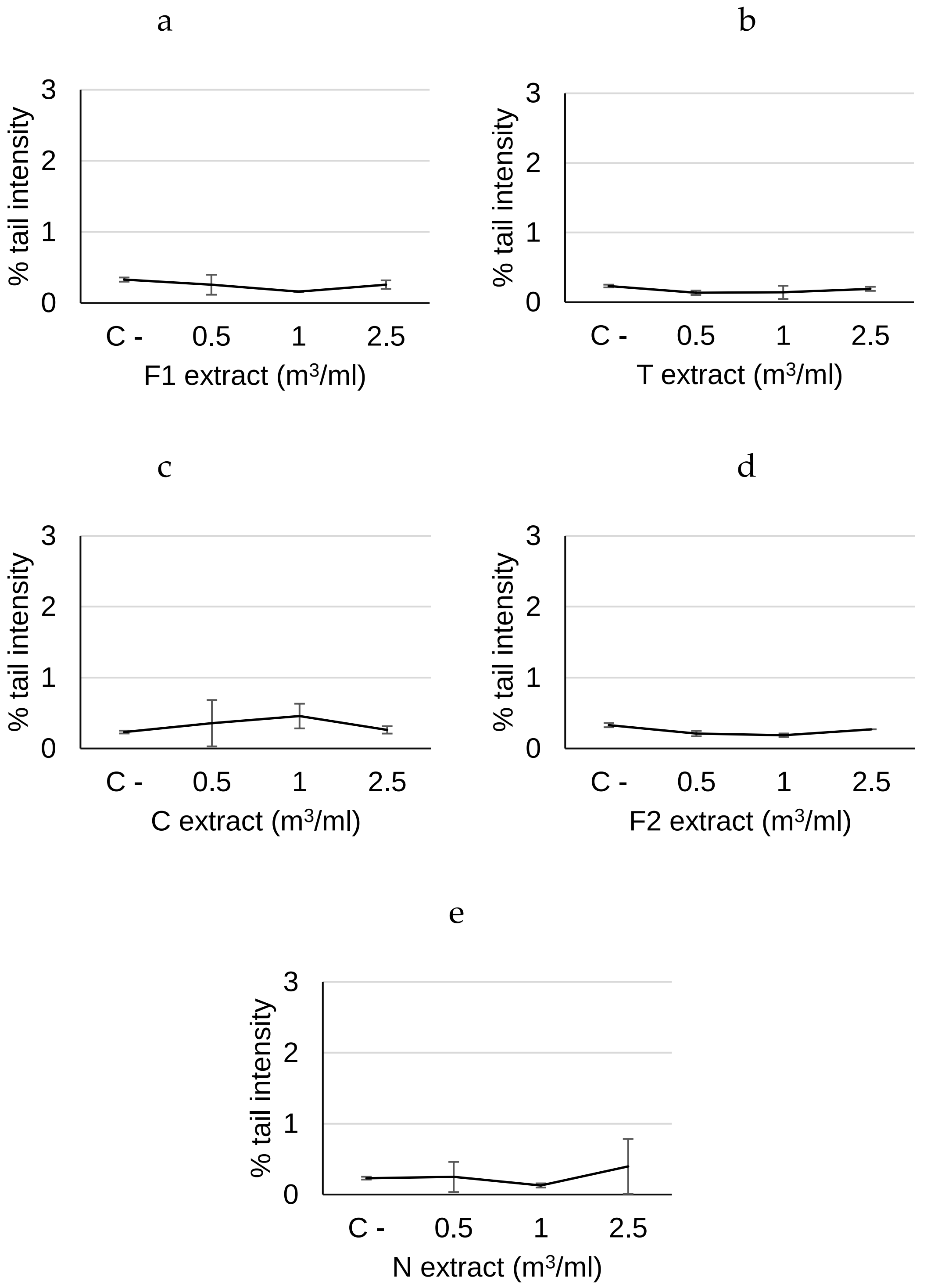

Figure S4. DNA damage detected using Comet assay in PM_{10} extracts collected in (**a**) Chiomonte (wildland fire) – F1; (**b**) Torino – T; (**c**) Ceresole Reale – C and in $PM_{2.5}$ extracts collected in (**d**) Chiomonte (wildland fire) – F2 (**e**) Novara – N. Data expressed as % of tail intensity (means and standard deviations). One-way ANOVA test followed by *post-hoc* Dunnett test not significant (data *vs* C-, $p > 0.05$).

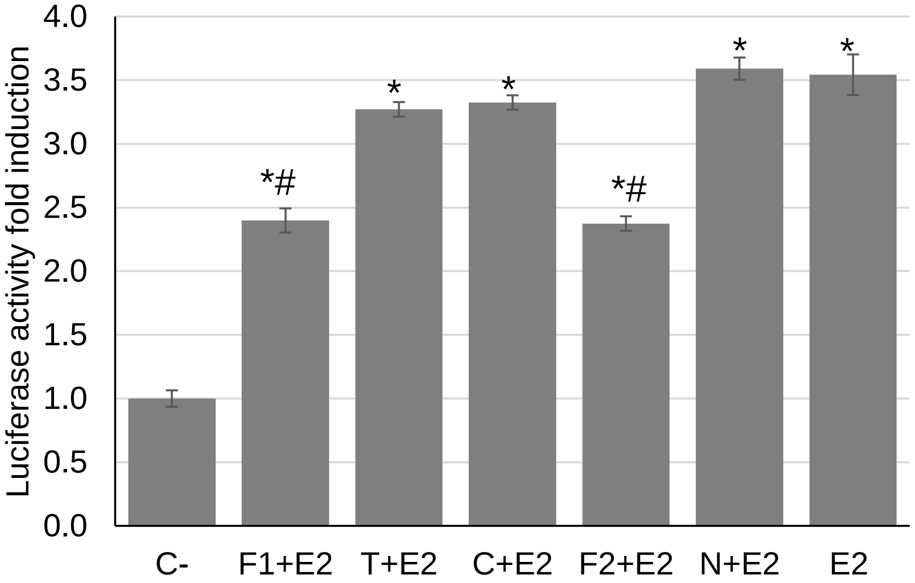

Figure S5. Estrogenic activity detected using luciferase gene reporter assay in the PM extracts (1 m^3/ml) tested in combination with 17β-estradiol (10^{-10} M). The results are expressed as luciferase activity fold induction (means and standard deviations) respect to negative control (C- =1). E2 = 17β-estradiol (10^{-10} M). *$p < 0.05$ *vs* C- according to T- test. #$p < 0.05$ *vs* E2 according to t-test.
